# Visual Cues of Object Properties Differentially Affect Anticipatory Planning of Digit Forces and Placement

**DOI:** 10.1371/journal.pone.0154033

**Published:** 2016-04-21

**Authors:** Trevor Lee-Miller, Michelle Marneweck, Marco Santello, Andrew M. Gordon

**Affiliations:** 1 Department of Biobehavioral Sciences, Teachers College, Columbia University, New York, New York, United States of America; 2 School of Biological and Health Systems Engineering, Arizona State University, Tempe, Arizona, United States of America; University of Exeter, UNITED KINGDOM

## Abstract

Studies on anticipatory planning of object manipulation showed initial task failure (i.e., object roll) when visual object shape cues are incongruent with other visual cues, such as weight distribution/density (e.g., symmetrically shaped object with an asymmetrical density). This suggests that shape cues override density cues. However, these studies typically only measured forces, with digit placement constrained. Recent evidence suggests that when digit placement is unconstrained, subjects modulate digit forces *and* placement. Thus, unconstrained digit placement might be modulated on initial trials (since it is an explicit process), but not forces (since it is an implicit process). We tested whether shape and density cues would differentially influence anticipatory planning of digit placement and forces during initial trials of a two-digit object manipulation task. Furthermore, we tested whether shape cues would override density cues when cues are incongruent. Subjects grasped and lifted an object with the aim of preventing roll. In Experiment 1, the object was symmetrically shaped, but with asymmetrical density (incongruent cues). In Experiment 2, the object was asymmetrical in shape and density (congruent cues). In Experiment 3, the object was asymmetrically shaped, but with symmetrical density (incongruent cues). Results showed differential modulation of digit placement and forces (modulation of load force but not placement), but only when shape and density cues were congruent. When shape and density cues were incongruent, we found collinear digit placement and symmetrical force sharing. This suggests that congruent and incongruent shape and density cues differentially influence anticipatory planning of digit forces and placement. Furthermore, shape cues do not always override density cues. A continuum of visual cues, such as those alluding to shape *and* density, need to be integrated.

## Introduction

Skilled object manipulation relies on precise anticipatory planning processes that in turn rely on visual estimates of object properties and prior grasping experience [1–3]. Specifically, visual cues of size, shape and density allow for inference of object properties, such as center of mass (CM) [4–6], which we use with sensorimotor memory representations obtained from prior grasping experience [7–9] to plan for skilled object manipulation.

Object properties inferred from visual shape cues may be different from actual object properties, such as an object with a symmetrical shape cue and an asymmetrical density distribution (e.g., inverted T-shaped object with one side of the base heavier than the other side). Studies investigating anticipatory planning processes in *initially* manipulating such objects have shown failed performance as indicated by an inability to maintain the vertical angle of the object in the frontal place (i.e., large object roll), even when subjects had knowledge of the density distribution [6,10–12]. For example, when presented with a symmetrical inverted T-shaped object consisting of a brass bar on the one side and a hollow plastic bar on the other side, subjects accurately estimated the location of the CM (by pointing). However, during initial lifts with such objects, instead of partitioning load forces to generate an appropriate compensatory moment (Mcom) in the opposite direction of the CM, subjects applied symmetrical load forces, resulting in a roll [6].

The results of the above studies [6,10] suggest that visual shape cues may *initially* override visual density (i.e. weight distribution) cues (when these cues are incongruous) during planning of manipulating such objects. However, these studies constrained digit placement to be collinear (i.e., on force sensors). It has recently been shown that unconstrained digit placement during manipulation of such objects (i.e., with incongruent shape and weight distribution) results in partitioning of *both* digit position and load forces (i.e., higher digits and forces on the heavier side) to generate torques to counter object roll [3,11,13,14]. Furthermore, we know from studies that investigate effects of rotation of these objects that appropriate digit placement is learned faster than digit force partitioning, although modulation of digit placement alone was not sufficient to prevent roll entirely [11]. Based on the faster learning of placement, the authors suggested that digit placement is an explicitly controlled process that is independent from digit forces that are implicitly controlled. Thus, it is conceivable that subjects could modulate digit placement, but not forces, during initial trials with objects such as that used in the Craje et al. study [6] (incongruent shape and weight distribution).

In the present study, we tested the overall hypotheses that shape and weight distribution (density) cues will differentially influence digit placement and forces (e.g., placement but not force modulation on initial lifts). Furthermore, we tested the hypothesis that shape cues will override weight distribution cues when these cues are incongruent. We first replicated the Craje et al. study [6] to determine whether symmetrical shape cues override density cues during initial manipulation, when the information they provide is incongruent. However, unlike that study, digit placement here was unconstrained. Next, we investigated whether anticipatory planning might be improved when shape and density cues are congruent (i.e. an L-shaped object with an asymmetric weight distribution), with digit placement unconstrained. This condition was similar to that tested in previous studies [10,15] which found subjects applied an Mcom to reduce object roll on the first trial, but the Mcom was of insufficient magnitude to prevent object roll. Again, here, unlike previous studies, we examined the contributions of *both* digit placement and forces in generating an Mcom that was appropriate to the object’s weight distribution. Lastly, we investigated anticipatory planning of manipulating an object with an asymmetric shape cue and a symmetric weight distribution cue (e.g., an inverted T-shaped object with equal lengths of brass on each side, but with hollow plastic extending one side of the T). This condition created incongruence unlike that tested in prior studies, since here the shape was asymmetrical whereas the CM was still at the object’s center. Thus, we tested whether asymmetrical shape cues would override symmetrical density cues (resulting in incorrect partitioning of digit placement and forces compared to correct symmetrical digit placement and forces).

## Materials and Methods

### Participants

Sixty healthy young adults (median age: 26 years, range: 20–37 years; 21 males), recruited between January 2014 and April 2014, participated in the study. Participants were right-handed, had normal or corrected-to-normal vision, and had no upper limb orthopedic impairments. Written informed consent was obtained prior to participation in compliance with the Declaration of Helsinki. The study was approved by the Teachers College, Columbia University Institutional Review Board.

### Apparatus

**Fig 1:**
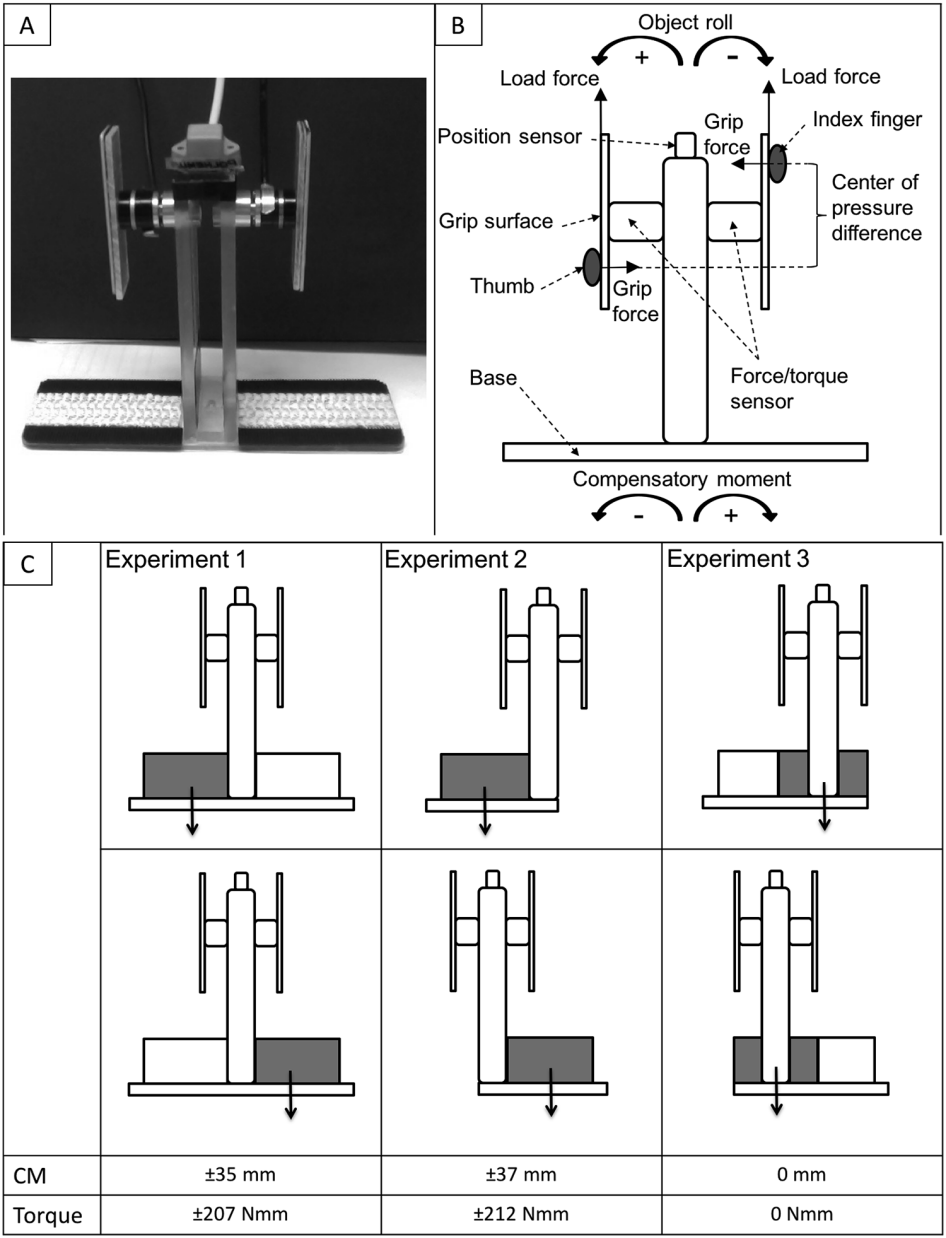
Experimental set-up. A. Custom-made grip device without weights, with the horizontal base used in Experiment 1. B. Schematic drawing of the grip device with a right-handed precision grip. C. Experimental configurations. Dark colored blocks represent metal weights and light colored blocks represent plastic weights. Arrows indicate actual CM location. CM was measured with respect to the center of the device. A positive value indicated CM toward the index finger side while a negative value indicated CM toward the thumb side. Torque indicated represents the moment generated by the CM at the center of the object.

A custom-made grip device with a central vertical column and 3 interchangeable horizontal bases was used to measure the torques and forces of the thumb and index finger (Fig 1). The central vertical column of the device consisted of two balsawood-covered plastic rectangular grip surfaces (height: 70 mm, width: 20 mm, thickness: 4 mm, distance between grip surfaces: 80 mm) attached to two multi-axis force sensors (Nano 17 Force/Torque transducer, ATI Industrial Automation, NC) that were mounted onto a Plexiglass block (height: 130 mm, width: 24 mm, depth: 40 mm). The force sensors measured grip force, load force, and torques applied (resolution = 0.05 N, 0.025 N, 0.125 Nmm respectively). Using a right-handed precision grip, the thumb could be placed anywhere on the left grip surface and the index finger could be placed anywhere on the right grip surface. An electromagnetic sensor (Polhemus Fasttrack, 0.005 mm of range, and 0.025° resolution) was mounted on top of the Plexiglas block to measure vertical position and roll of the grip device.

The congruency of visual cues of object shape and object density distribution was manipulated to probe its effect on anticipatory planning of digit placement and forces during initial trials. To manipulate visual cues of object shape, 3 interchangeable horizontal bases were made of Plexiglass with different lengths (180 mm, 130 mm, 105 mm, respectively). Each base could be attached to the central vertical column resulting in the three differentially shaped objects (see Fig 1): a visually symmetrical inverted T-shaped object, a visually asymmetrical L-shaped object, and a visually asymmetrical inverted T-shaped object with one end shorter than the other. To manipulate visual cues of object density distribution and thus CM, we used 4 different weights (height: 25 mm, depth: 25 mm): a solid metal bar (width: 75 mm, mass: 405 g), a hollow plastic bar (width: 75 mm, mass: 10 g), a solid metal cube (width: 25 mm, mass: 135 g), and a solid metal cube affixed to a hollow plastic bar (width: 75mm, mass: 140 g). These weights were placed on the horizontal bases of the object resulting in three different configurations (Fig 1c). The first configuration, used in Experiment 1, a symmetrical inverted T-shape with asymmetrical density distribution, was arranged with the metal bar on the thumb side with the plastic bar on the index finger side. The CM of this configuration was on the side of the metal bar (thumb). The second configuration, used in Experiment 2, an asymmetrical L-shape with asymmetrical density, was arranged with the solid metal bar on the thumb side of the base. The CM of this configuration was on the side of the metal bar (thumb). The third configuration, used in Experiment 3, an asymmetrical inverted T-shape with symmetrical density, was arranged with the solid metal cube on the shorter side of the base (index finger) and the solid metal cube and hollow plastic bar on the longer side of the base (thumb). The CM of this configuration was the center of the device. Therefore, the configurations in each the three experiments, varied in their shape and density arrangement. The configuration in Experiment 1 resulted in incongruence between visual cues of object shape and visual cues of density distribution where shape cues indicated a centered CM while density cues indicated an off-centered CM (towards the thumb/left side). The configuration in Experiment 2 resulted in congruence between visual cues of object shape and visual cues of density distribution where both indicated an off-centered CM (towards the thumb/left side). The configuration in Experiment 3 resulted in incongruence between visual cues of object shape and visual cues of density distribution where shape cues indicated an off-centered CM (towards the thumb/left side) while density cues indicated a centered CM.

Each experiment had two conditions. In Experiment 1 and 2, the CM location was on the left in one condition and on the right in the other condition. In Experiment 3, the shape cue (the longer base-side) was on the left in one condition and on the right in the other condition. A possible limitation of our previous studies [6] was that all participants experienced all conditions. Thus, even if the data did not suggest it, there could have been proactive interference preventing appropriate force scaling. To prevent this, in this study, participants performed only one condition (condition 1 or 2 in either Experiment 1, 2, or 3), with ten participants per condition.

### Procedure

Participants were seated in front of a height-adjustable table, with their right elbow flexed 90° in the parasagittal plane, with their right shoulder in line with the midpoint of the object, and their right hand on a start marker on the table. Participants performed a hefting task of the individual parts, then a CM estimation task of the object with its shape and density configuration in place. The object was then placed 20 cm from a start marker at the end of the table. Following this was the lifting task where the goal was to lift the object while minimizing object roll. Participants performed 10 lifts in total following which they were asked to again estimate the CM.

#### Hefting and CM estimation task

Results from our previous study [6] showed that hefting the individual parts of the object separately did not improve performance during initial trials. However, to ensure that participants had explicit knowledge of the density of the different weights, the hefting task was performed. Prior to estimating the CM of the object, the participant hefted the device, and the respective weights separately. Following this hefting procedure, the participant was presented with the device in the desired configuration. The participant was then asked to indicate where he/she thought the horizontal CM of the object was, by pointing with a pen on a ruler placed in front of the object. This CM estimation task was done to determine whether participants could use visual cues of object shape and density distribution to estimate the CM of the object. At the end of the 10 trials, the participant again estimated the CM of the object to test if experience lifting the object modified their CM estimation [9,13,14].

#### Lifting task

After the CM estimation task, the participant was asked to place their right hand (palm down) on the start marker. The task goal was to minimize object roll. The participant was instructed to lift the object in a smooth self-directed manner. For each trial, following an audio tone, the participant reached and grasped the object using his/her right thumb and index finger, anywhere on the respective grip surfaces, and lifted the object vertically upwards to the height of a 15-cm marker placed next to the object. The participant held the object at that height until presentation of a second audio tone (5s after first tone), after which he/she placed the object back on the table, and returned his/her hand to the start marker awaiting the start of the next trial.

### Data Processing

Throughout the lifts, digit forces and torques applied to the grip surfaces recorded by the force transducers, and position data recorded by the electromagnetic sensor were sampled at 500 and 120 Hz, respectively, using custom written software in WinSC and then analyzed in WinZoom (Umeå University, Sweden). Data collected were filtered using a second-order low pass Butterworth filter with a cutoff of 6 Hz. Lift onset was defined as the point at which the vertical position of the object went above 1 mm and subsequently remained above this value. Object roll occurred in the parafrontal plane and all variables involved forces within this plane. The outcome measures included:

1. Peak object roll, defined as the angle of the object in the frontal plane. Peak object roll was recorded shortly (< 500 ms) after lift onset before somatosensory feedback resulted in corrective responses to counter object roll. Positive values represent counterclockwise roll (towards the thumb) and negative values represent clockwise roll (towards the index finger).
2. Digit load force (LF) at lift onset is the tangential component of the force produced by each digit measured in newton (N).
  - Load force difference (*LF*_*diff*_) = *LF*_*thumb*_ − *LF*_*index*_
    Positive values indicate larger thumb than index finger load force while negative values indicate larger index finger than thumb load force.
3. Digit center of pressure (COP) is the measure of digit placement defined as the point of contact of each digit on the grip surface measured in millimeter (mm). This was computed using the formula:
  $$COP_{digit}=\left(\;Tx_{digit}-\left(LF_{digit}\;\times \;thickness\;of\;grip\;surface\right)\right)\;∕\;GF_{digit}$$
  where *Tx*, digit torque in the frontal plane, is the torque generated by each digit on the grip surface measured in newton millimeter (Nmm). In this instance, the grip surface was the lever arm while the force transducer was the fulcrum. The thickness of the grip surface was 4 mm, and GF, the digit grip force at lift onset, is the normal component of the force produced by each digit measured in newton (N).
  - Center of pressure difference (*COP*_*diff*_) = *COP*_*thumb*_ − *COP*_*index*_
    Positive values indicate higher thumb than index finger COP while negative values indicated higher index finger than thumb COP.
4. Compensatory moment (Mcom) defined as the anticipatory moment generated by the digits in response to the representation of object CM measured in newton millimeter (Nmm). This was computed using the formula:
  $$Mcom\;=\;\left(LF_{diff}\;\times \;\frac{d}{2}\right)\;+\;(\;GF_{mean}\;\times \;COP_{diff})$$
  where *d* is the width between both grip surfaces (80 mm). A positive Mcom represented a clockwise moment while a negative Mcom represented a counter-clockwise moment.

### Data Analysis

Peak roll was used to determine accomplishment of task goal. Anticipatory planning of digit forces and placement were analyzed using the resultant Mcom, digit COP and digit load force at lift onset.

#### Effect of shape and density cues on CM estimation

We performed mixed ANOVAs with trial (before lift 1 and after lift 10) as the within-subjects factor and experimental configuration (Experiment 1, 2, and 3) as the between-subjects factor, with the CM location (Experiment 1 and 2) and shape cue (Experiment 3) on the left and right, respectively.

#### Effect of shape and density cues on anticipatory planning

For Experiments 1 & 2, we performed mixed ANOVAs with trial (lift 1 and lift 10) as the within-subjects factor and CM location (left and right side) as the between-subjects factor on the peak roll, Mcom, COP difference, and load force difference. For Experiment 3, we performed mixed ANOVAs with trial (lift 1 and lift 10) as the within-subjects factor and shape cue (left and right side) as the between-subjects factor on each of each measure. Interaction effects were examined using tests of simple main effects. Bonferroni corrections were used where applicable. Significance was considered at the *p* < .05 level.

## Results

### CM Estimation

**Fig 2:**
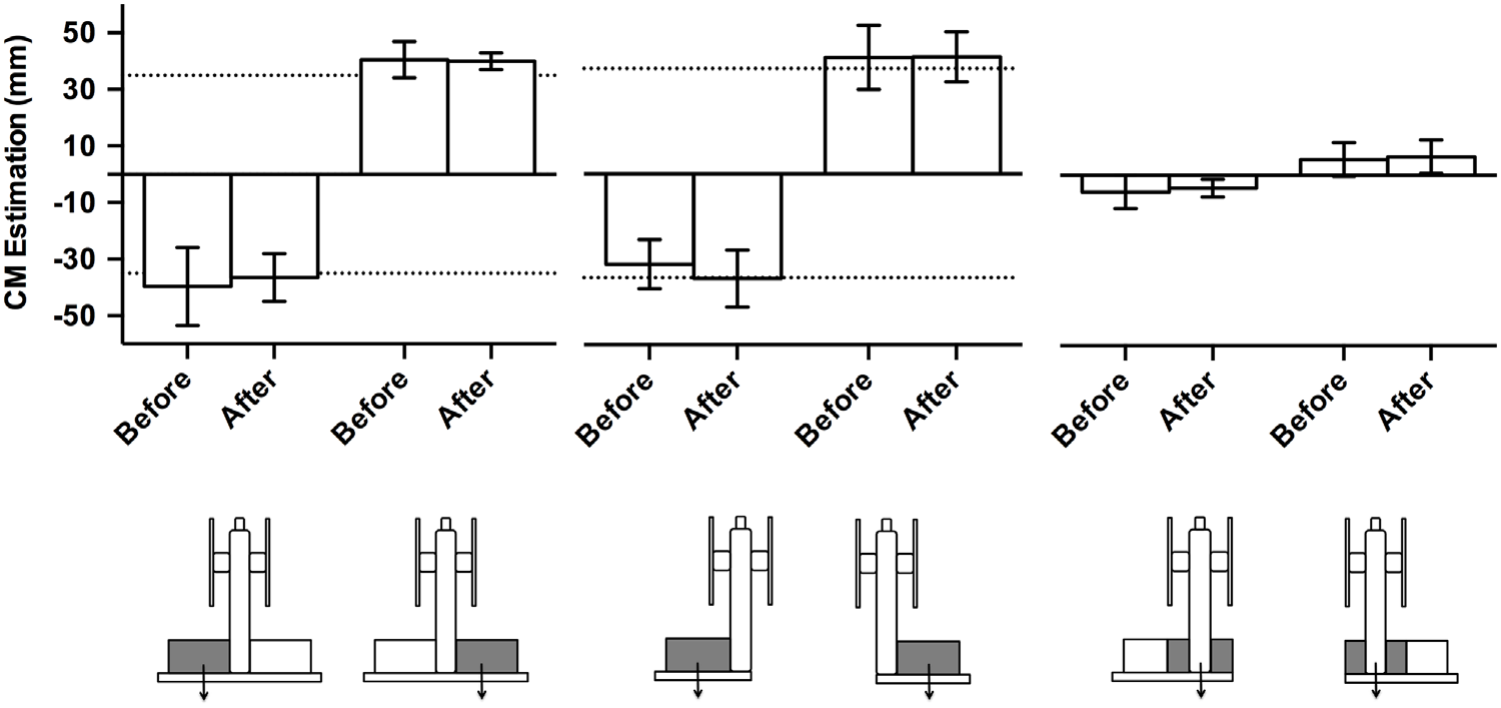
Center of mass estimation. Mean CM estimations of configurations in the 3 experiments (Experiment 1: left panel; Experiment 2: middle panel; Experiment 3: right panel) measured as the distance from the central column before and after lifting. Zero represents the midline of the central column, negative values represent the CM estimate on the thumb side, and positive values represent the CM estimate on the index finger side. Dotted lines indicate actual CM of object. The CM of the configuration in Experiment 3 is zero. Error bars represent upper and lower limit of 95% confidence interval.

Participants in each of the experiments accurately estimated the CM location before and after lifting the object (+/- 5 mm from the CM location, see Fig 2, with no significant differences in CM estimation before and after lifting and no interaction between trial and experiment configuration, *p* > .05 in all cases). Thus, participants were able to use visual density cues to estimate the CM location accurately for each of the object configurations.

### Experiment 1: Symmetrical shape and asymmetrical density cues

**Fig 3:**
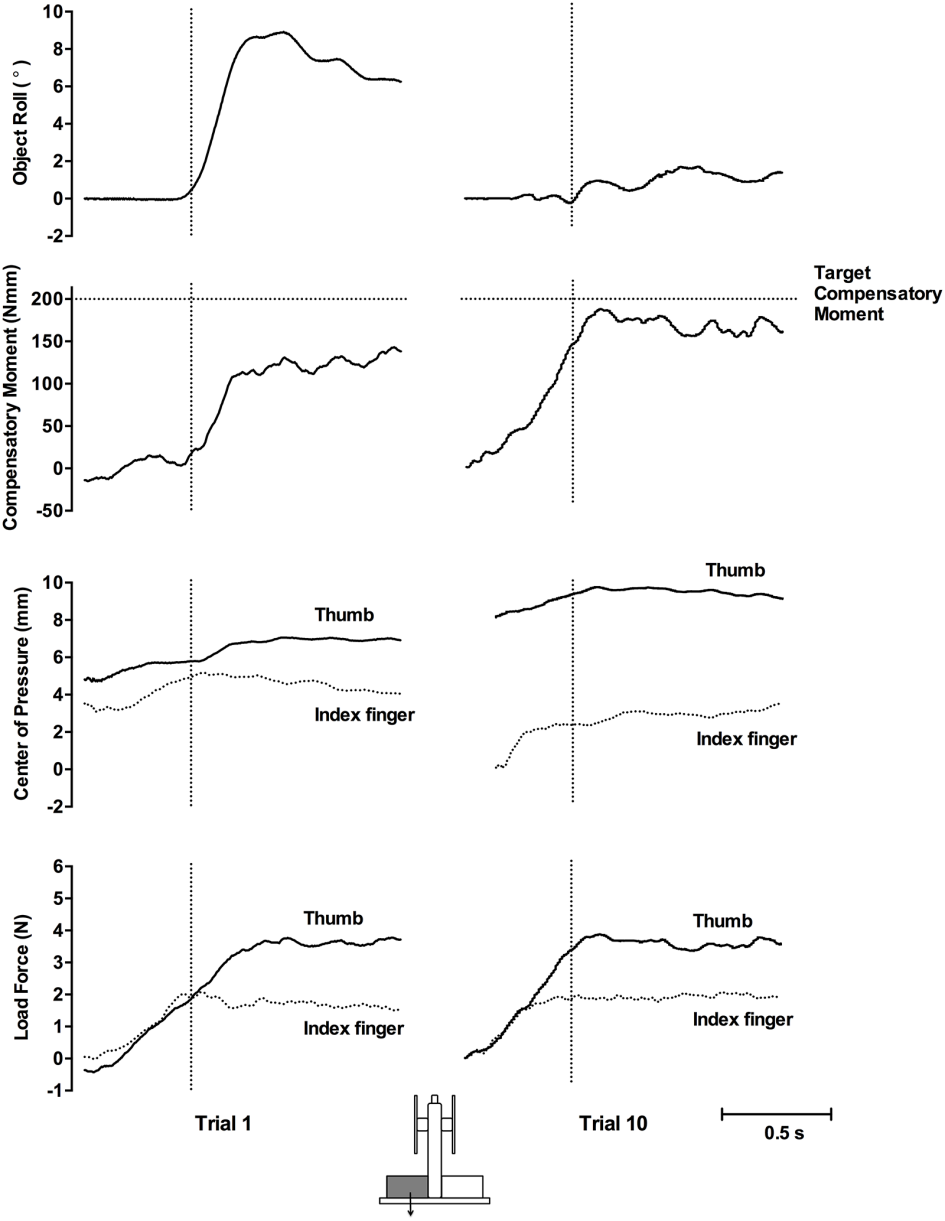
Representative plots, Experiment 1. Plots show trial 1 (left panel) and trial 10 (right panel) in the left CM condition. Vertical dotted lines indicate lift onset. Horizontal dotted lines in the second row show the target Mcom required to equal object torque. In the COP and load force rows, solid lines represent the thumb and dotted lines represent the index finger.

Fig 3 shows the object roll, Mcom, COP, and load force of a representative participant for the first and tenth lifts of the inverted T-shaped object with a left CM during Experiment 1. On the first lift, there was a large peak roll due to the negligible Mcom generated by the participant, which was insufficient to counter the torque of the object. The small Mcom was caused by the very small COP difference, with the thumb only slightly higher than the index finger, and symmetrical thumb and index finger load force. Comparatively, on the tenth lift, there was a small roll owing to the large Mcom generated. This Mcom was a result of both a higher thumb than index finger COP and a larger thumb than index finger load force.

**Fig 4:**
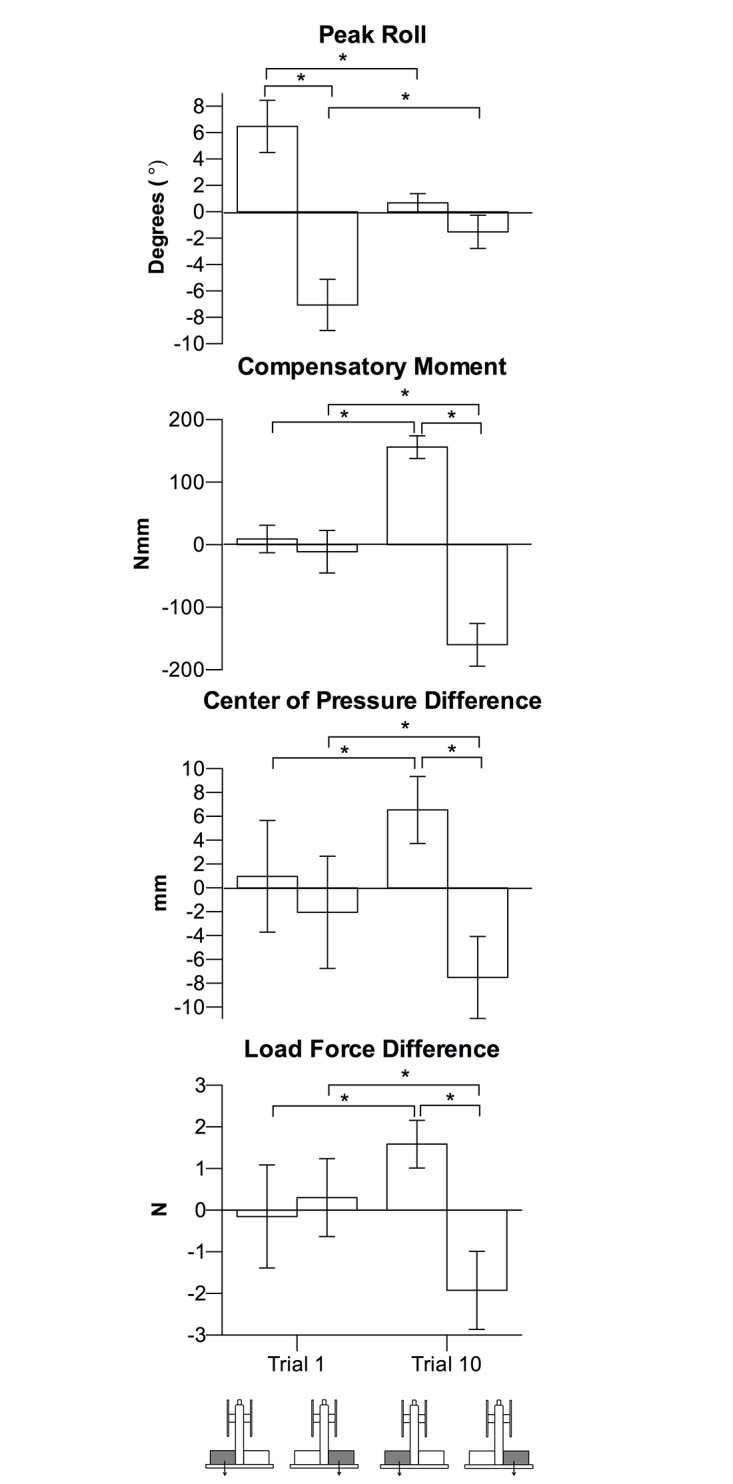
Experiment 1 lifting task results. Group means (95% confidence interval) at trial 1 and trial 10 of peak roll, Mcom, COP difference, and load force difference in left and right CM conditions. Asterisks indicate *p* < .05.

As described below, and seen in Fig 4, group means between the left and right CM locations and between trial 1 and trial 10 were generally consistent with this representative illustration. There was a trial x CM location interaction for peak roll, *F*(1, 18) = 84.51, *p* < .001, η_p_^2^ = .82), with larger rolls in the direction of the CM location at trial 1 than at trial 10 (*p’s* < .05). Peak roll at trial 1 was due to a lack of Mcom by participants (trial x CM location, *F*(1, 18) = 159.06, *p* < .001, η_p_^2^ = .90). Specifically, there was no difference between the Mcom for the left and right CM location at trial 1 (*p >*.*05*), but at trial 10, an appropriate Mcom was generated (of opposing direction between CM locations, *p* < .05).

The lack of Mcom at trial 1 was a result of collinear digit placement and digit load forces. Specifically, there was a trial x CM location interaction for COP difference (*F*(1, 18) = 14.98, *p* < .001, η_p_^2^ = .45), where participants did not exhibit different digit placements between the left and right CM locations at trial 1 (*p* > .05), but did so at trial 10 (*p* < .05). Similarly, there was a trial x CM location interaction for load force difference (*F*(1, 18) = 27.45, *p* < .001, η_p_^2^ = .60), where participants did not partition load force according to CM location at trial 1 (*p* > .05), but did so at trial 10 (*p* < .05).

### Experiment 2: Asymmetrical shape and asymmetrical density cues

**Fig 5:**
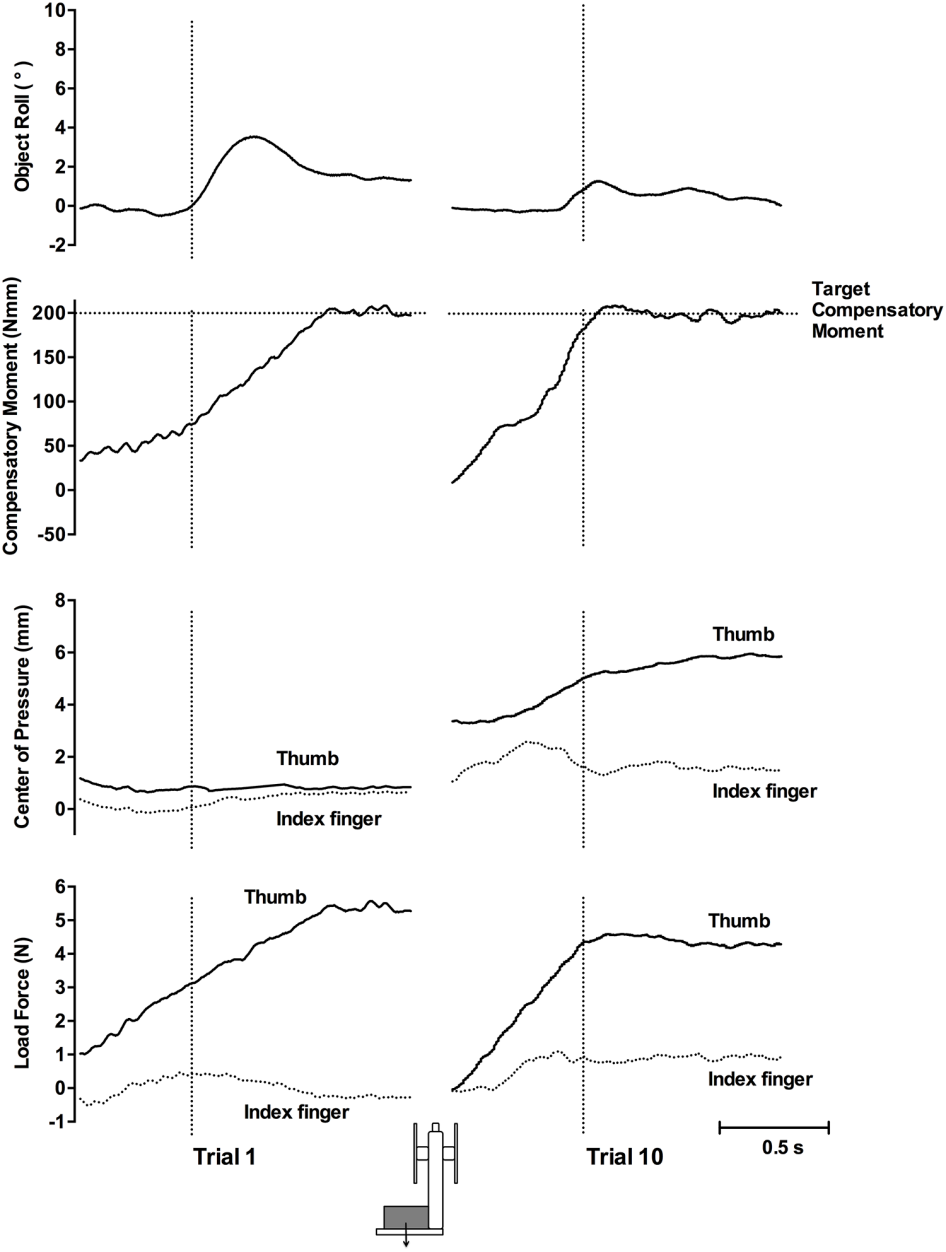
Representative plots, Experiment 2. Plots show trial 1 (left panel) and trial 10 (right panel) in the left CM condition. Vertical dotted lines indicate lift onset. Horizontal dotted lines in the second row show the target Mcom required to equal object torque. In the COP and load force rows, solid lines represent the thumb and dotted lines represent the index finger.

The previous experiment requiring manipulation of an object with an incongruent shape and density cue showed erroneous anticipatory planning of both digit placement and load forces. Here we tested whether congruent shape and density cues (i.e., an L-shaped object with a uniform density) would result in modulation of digit placement and forces to achieve an appropriate Mcom. Fig 5 shows the object roll, Mcom, COP, and load force of a representative participant for the first and tenth lifts with the object’s CM on the left during Experiment 2. On the first lift, there was a large peak roll. This large roll was due to an insufficient Mcom generated by the participant. However, the Mcom generated was in the appropriate direction to counter object torque. There was a very small COP difference, with the thumb only slightly higher than the index finger, but the Mcom was the result of the larger thumb to index finger load force. The participant could infer object CM, and an Mcom in the appropriate direction was generated, however, this did not prevent object roll. Comparatively, on the tenth lift, there was a small roll owing to a large Mcom generated. This Mcom was a result of both a higher thumb to index finger COP and a larger thumb to index finger load force.

**Fig 6:**
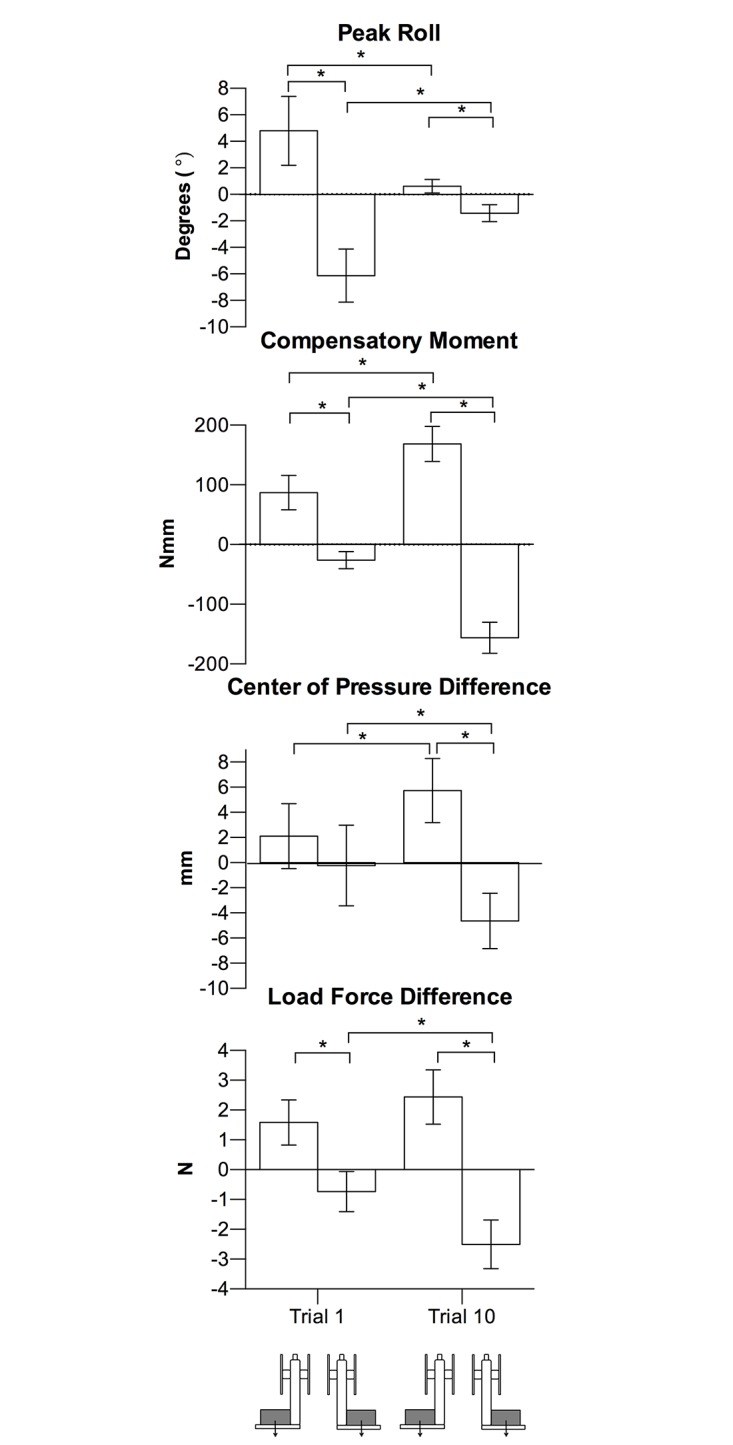
Experiment 2 lifting task results. Group means (95% confidence interval) at trial 1 and trial 10 of peak roll, Mcom, COP difference, and load force difference in left and right CM conditions. Asterisks indicate *p* < .05.

As described below, and seen in Fig 6, group means between the left and right CM locations and between trial 1 and trial 10 were generally consistent with this representative illustration. There was a trial x CM location interaction for peak roll (*F*(1, 18) = 45.03, *p* < .001, η_p_^2^ = .71), with larger rolls in the direction of the CM location at trial 1 than at trial 10 (*p’s* < .05). Unlike in Experiment 1, the Mcoms were different (and in the correct direction) between the left and right CM locations on trial 1 (*p* < .05). However, the differences (*p* < .05) were larger at trial 10 (trial x CM location interaction, *F*(1, 18) = 159.61, *p* < .001, η_p_^2^ = .90).

The lack of Mcom of appropriate magnitude to prevent large roll at trial 1 was a result of collinear digit placement. Specifically, there was a trial x CM location interaction for COP difference *F*(1, 18) = 12.34, *p* < .05, η_p_^2^ = .41), where participants did not exhibit different digit placements between the left and right CM locations at trial 1 (*p* > .05), but did so at trial 10 (*p* < .05). The load force partitioning between digits were different (and in the correct direction) between the left and right CM locations on trial 1 (*p* < .05), but the difference (*p* < .05) was larger at trial 10 (trial x CM location interaction, *F*(1, 18) = 15.99, *p* < .05, η_p_^2^ = .47).

### Experiment 3: Asymmetrical shape and symmetrical density cues

Overall, the previous experiment requiring manipulation of an object with congruent asymmetrical visual shape and density cues resulted in the modulation of Mcom by modulating digit load force (but not digit placement). The resultant effect was insufficient to prevent object roll.

**Fig 7:**
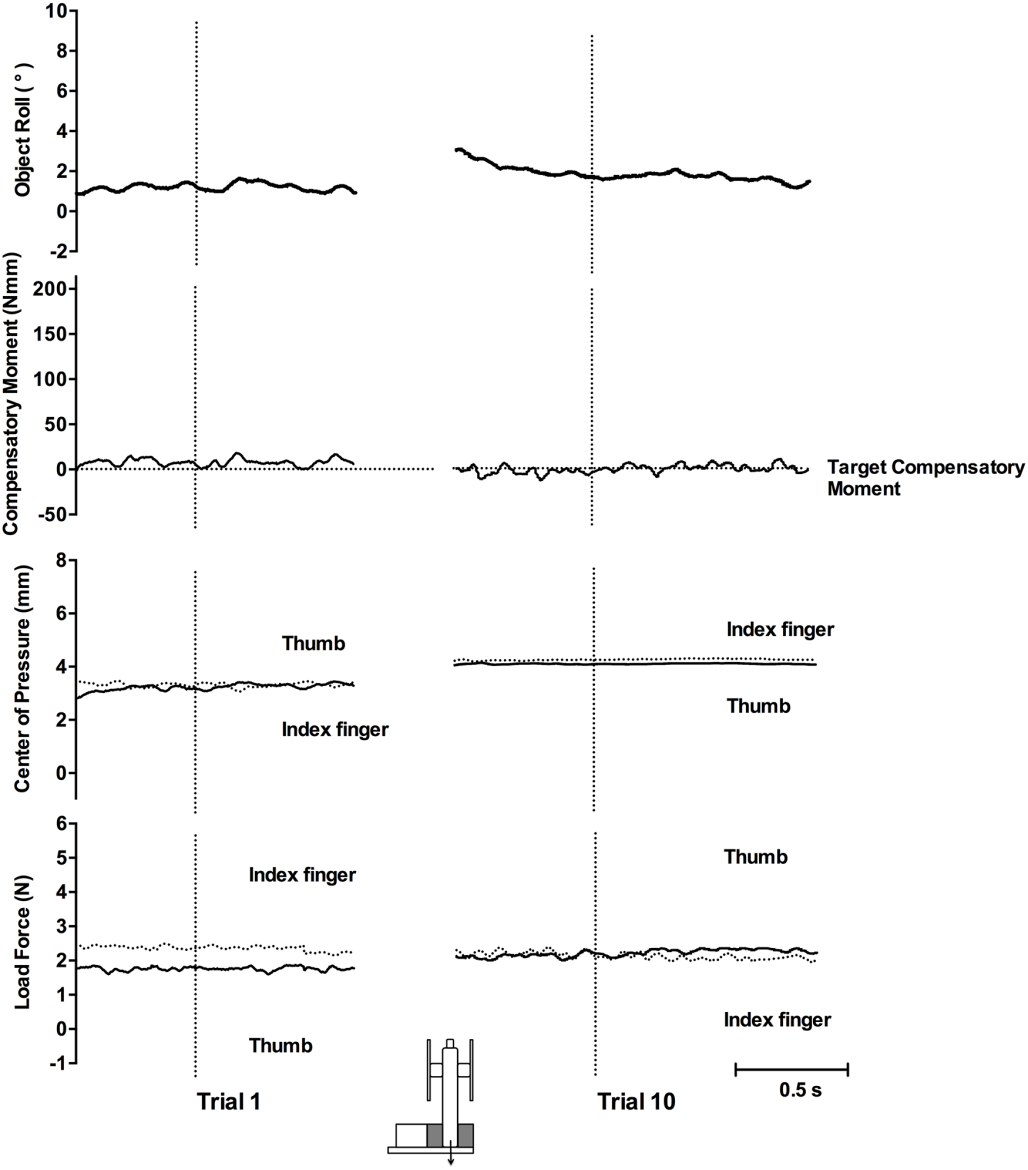
Representative plots, Experiment 3. Plots show trial 1 (left panel) and trial 10 (right panel) in the left shape cue condition. Vertical dotted lines indicate lift onset. Horizontal dotted lines in the second row show the target Mcom required to equal object torque. In the COP and load force rows, solid lines represent the thumb and dotted lines represent the index finger.

To determine whether the results of the prior two experiments (and prior studies) are the result of visual shape cues overriding weight distribution (density) cues, here we present subjects with an asymmetrically-shaped object with weight distribution cues indicating a centered CM. Fig 7 shows the of object roll, Mcom, COP, and load force of a representative participant for the first and tenth lifts with the object’s CM on the left during Experiment 3. On the first and tenth lift, object roll was minimal, Mcom generated was small, and digit COP and load forces were collinear throughout.

**Fig 8:**
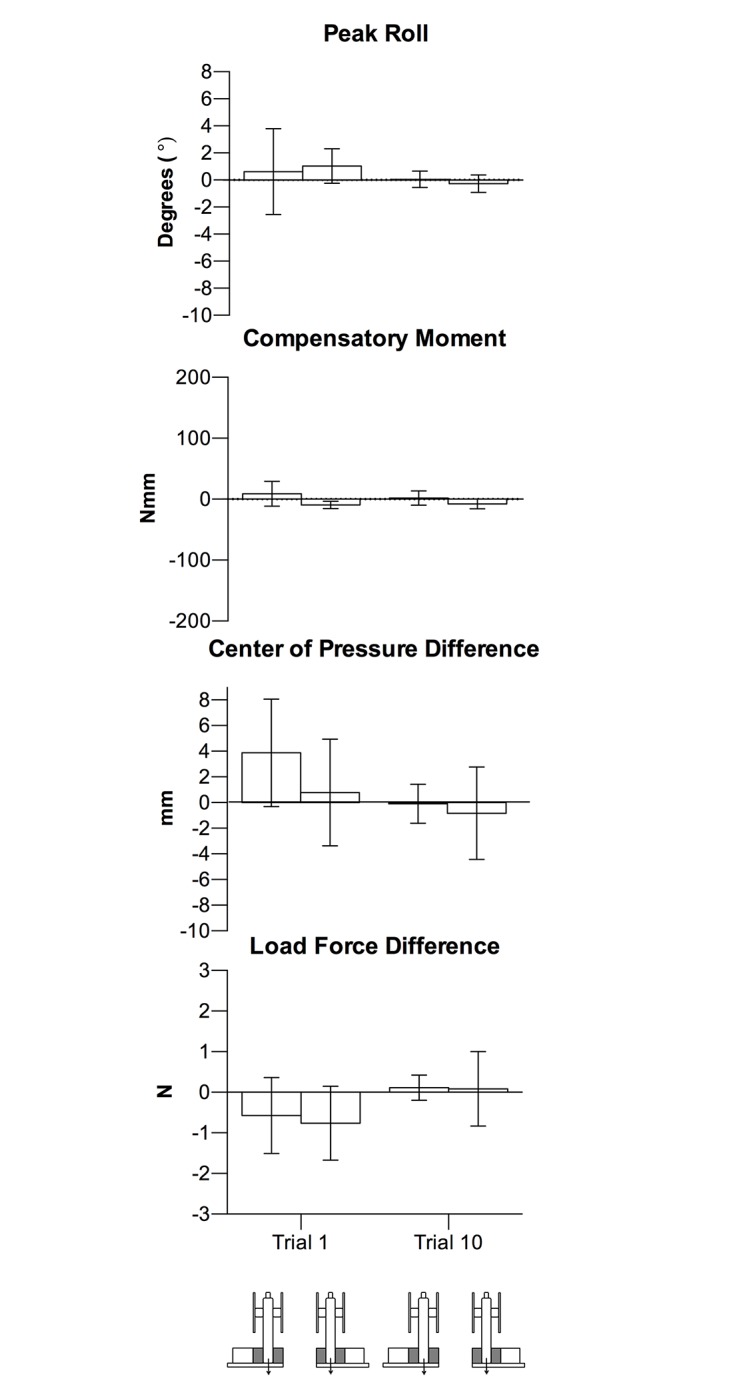
Experiment 3 lifting task results. Group means (95% confidence interval) at trial 1 and trial 10 of peak roll, Mcom, COP difference, and load force difference in the left and right shape cue conditions.

As seen in Fig 8, the asymmetrical shape cue did not result in erroneous object roll (*p’s* > .05) and participants generated minimal Mcom, with no Mcom differences between the left and right shape cues at both trials 1 and 10 (*p’s >*.05). The lack of Mcom difference was a result of nearly collinear digit placement and digit load forces for both left and right shape cues. Participants did not exhibit different digit placement and forces between left and right shape cues at both trials 1 and 10, with no significant interaction between trial and shape cue (*p* > .05).

## Discussion

The present study investigated the effect of congruent and incongruent visual cues of shape and weight distribution (density) on anticipatory planning of digit placement and forces. Our first hypothesis, testing whether shape and density cues differentially influenced digit placement and forces on initial trials, was partially supported. In line with our hypothesis, we found differential modulation of digit placement and forces on initial trials, but only when shape and density cues were congruent. However, in contrast to our hypothesis, we found that load force, instead of digit placement, was modulated and contributing to an Mcom in the correct direction. In testing our second hypothesis, whether shape cues will override density cues when cues are incongruent, our results showed this to not be the case in all instances. Specifically, when shape and density cues were incongruent, subjects adopted collinear digit placement and forces. This resulted in an inappropriate Mcom in Experiment 1 (i.e., a symmetrical inverted T-shape with asymmetrical weight distribution), but led to an appropriate Mcom in Experiment 3 (i.e., a centered weight distribution). We discuss these findings in relation to previous work and possible motor planning mechanisms.

### Incongruent cues lead to symmetrical digit placement and forces

Our previous work has identified a dichotomy between the ability to accurately estimate CM location and the use of that information to update or generate an internal model that would allow appropriate planning of digit forces [6]. Specifically, an object’s CM could be accurately estimated based on density cues, and subjects could use such density cues to scale the overall digit force, but only when the objects had consistent densities (i.e., they used higher forces on initial lifts with brass than plastic objects). However, on initial lifts with objects with inconsistent densities (i.e., CM location is off-centered), planning of digit forces did not result in generation of Mcom to counter object torque. Consistent with and adding to that finding, here we showed that this is not only present for the anticipatory planning of digit forces on initial trials, but also for the anticipatory planning of digit placement. Additionally, our results showed that the lack of force scaling on initial lifts reported in that study [6] was not due to proactive interference as the results were replicated in the present study using a between-subject design.

The results of Experiment 1 (symmetrical inverted-T shape with off-centered CM) indicated a possibility where the symmetrical shape cues, as opposed to the asymmetrical density cues, affected digit placement and forces (i.e., resulting in collinear positions and forces). It is unclear if the collinear pattern was due to the incongruent cues or preference for visual shape cues. To attempt to resolve between these possibilities, Experiment 3 involved lifts with an asymmetrical inverted-T shape with a centered CM. If visual shape cues were more robust in determining anticipatory planning, we would expect generation of an Mcom in the opposite direction of the longer arm. However, a lack of Mcom indicated that participants did not use the asymmetrical shape cue in the planning of the grasp task. There are two possible explanations for these findings from these two experiments. First, it could be that visual shape cues of symmetrical objects override other weight-distribution cues, whereas asymmetrical objects do not. This might be because we experience manipulations with many symmetrical objects in our daily lives, most of which have a symmetrical weight distribution (i.e., consistent material throughout). Second, when shape cues and density cues are incongruent, subjects might default to a behavior similar to when lifting symmetrically shaped objects where anticipatory planning of forces and placement are collinear. This would result in inappropriate Mcoms in Experiment 1, but appropriate Mcoms in Experiment 3. We cannot distinguish between these two possibilities. However, both possibilities also explain the failed transfer of digit placement and forces following object rotation and translation when the CM is off-centered but the object is symmetrical in appearance [10–12,15–19].

### Congruent cues lead to differential digit force modulation

Consistent with previous findings using objects with congruent shape and density cues, congruent cues in Experiment 2 (i.e., asymmetrical shape and densities) resulted in the generation of an Mcom that is in the appropriate direction required to reduce object roll [15]. Extending this finding, we have shown that the Mcom is a result of differential digit force modulation, and not digit placement modulation. This suggests that like visual size cues [20], visual shape cues with objects of consistent densities exert a powerful influence on grasp control during initial lifts. Thus, the findings of Experiment 3 are not due to subjects’ general inability to transform visual shape cues into a grasp performance, as they did so here, albeit when the object had a consistent density.

### Digit placement is not partitioned regardless of visual cues

Our data show that on initial trials, even with knowledge of object CM through visual shape and density cues, the thumb and index finger are placed collinearly to each other on either side. This is in contrary to our hypothesis that digit placement would be partitioned, which was based on previous findings that finger placement was more robust during memory retrieval of prior lifts compared to digit forces [11], suggesting not only differential learning rates but separate control mechanisms. Here, we show that visual shape and density cues are insufficient to elicit digit placement partitioning on the first trial in a series of (blocked) trials. This suggests that, when implicit knowledge of object properties can be obtained through subsequent lifts, digit placement is a context-dependent learned strategy where digit placement is planned only after obtaining knowledge of object torque through prior experience. Prior to this, a default collinear placement pattern is used. This strategy could have been used to prevent erroneous Mcom from being generated. Because of the strong association between digit force and placement [3], altering digit placement would be associated with altering digit forces. Without specific sensorimotor memories of the object, varying both forces and placement would lead to a large probability of making errors. Digit placement that is not collinear would result in the system having to adjust for digit force partitioning through further force modulation. Digit placement does not change after contact is made with the object, as such, placing the digits non-collinearly would create greater biomechanical constraints if the placement were wrong. For example, if the thumb were placed higher than the index finger when the object CM was on the right side, the index finger would have to apply a much larger load force in order to prevent object roll. Thus, the internal model might be set up in a default manner where the digits are placed collinearly, resulting in Mcom generation that is entirely due to force modulation. Another reason that could account for the little to no digit placement modulation in our study is that the torque used here is smaller than that used in previous work [14,15]. This could have contributed to participants resorting to a ‘digit force modulation’ only strategy, as opposed to a ‘digit force and position’ modulation strategy on the first lift. Nevertheless, our study suggests that when visual cues are incongruent, the complexity results in an error-based learning strategy where initial trials are used to gain implicit knowledge of object dynamics [21]. Thus, on initial trials, the default strategy of collinear digit forces and placement is used. When visual cues are congruent, with digit placement planned collinearly, digit forces are then anticipated and modulated in an attempt to generate an Mcom opposing object torque. It should be noted that predictability of an upcoming visual cue also may play a role in subjects’ ability or choice to modulate digit placement in response to that cue [2].

### Possible cortical networks involved in planning of digit forces and placement

Studies that have used repetitive transcranial magnetic stimulation (rTMS) inducing temporary virtual lesions on specific cortical regions have identified a fronto-parietal network of regions involved in the visuomotor control of digit forces and placement (when they are collinear). This network includes the anterior intraparietal area (AIP) [22], the dorsal and ventral premotor cortex (PMv) [23], and the primary motor cortex [24]. AIP and PMv have shown specifically to be involved in digit placement (with artificial lesions resulting in non-collinear digit placement). Furthermore, the above study [22] showed rTMS applied 270–220 ms before digit contact altered digit placement, whereas rTMS applied later, 170–120 ms before the digit contact, altered force scaling [22]. This suggests that AIP processes digit placement before force scaling. Thus, it supports our supposition that load force modulation (seen in Experiment 2) is based on collinear digit placement (because force scaling is processed after digit placement in AIP).

Regarding cortical networks that are involved in processing of visual cues of shape and density, sensory areas in the parietal and temporal cortices have been suggested to process visual cues of size and/or shape. Specifically, studies have pointed to AIP [25,26] and the superior parietal occipital cortex [27,28] in processing visual cues of object shape. These studies typically used objects with uniform densities. Moreover, these studies have assumed congruence between visual shape and density cues. To the best of our knowledge, the cortical regions involved in integration of object properties obtained through visual cues, such as shape and density, remains an untested question. Studies investigating potentially separate neural mechanisms responsible for processing visual cues of shape and density might give additional insights into how the system attempts to reconcile incongruent cues.

### Conclusions

We investigated the effect of congruent and incongruent visual cues of object shape and weight distribution (density) on anticipatory planning of digit placement and forces. With congruence between visual cues of object shape and density, we showed differential modulation of forces and placement, specifically, modulation of load force but not placement. With incongruence between visual cues of object shape and density, we showed collinear application of digit placement and symmetrical partitioning of forces. This suggests that congruent and incongruent visual cues of shape and density have a differential influence on anticipatory planning of digit forces and placement. A continuum of visual cues, such as those alluding to shape *and* density, need to be integrated. Such integration becomes more challenging when cues are incongruent, in which case the system defaults to a collinear force and positioning pattern. This happens even when the shape cue warrants non-collinear force and position.

## Supporting Information

S1 File Individual data points for Experiment 1, 2, and 3. (XLSX)
